# Human Dental Pulp Mesenchymal Stem Cell-Derived Soluble Factors Combined with a Nanostructured Scaffold Support the Generation of a Vascular Network In Vivo

**DOI:** 10.3390/nano13172479

**Published:** 2023-09-02

**Authors:** Ludovica Barone, Matteo Gallazzi, Federica Rossi, Roberto Papait, Mario Raspanti, Piero Antonio Zecca, Luca Buonarrivo, Barbara Bassani, Giovanni Bernardini, Antonino Bruno, Rosalba Gornati

**Affiliations:** 1 Department of Biotechnology and Life Sciences, University of Insubria, 21100 Varese, Italy; lbarone1@uninsubria.it (L.B.); federica.rossi@uninsubria.it (F.R.); roberto.papait@uninsubria.it (R.P.); l.buonarrivo@uninsubria.it (L.B.); giovanni.bernardini@uninsubria.it (G.B.); 2 Laboratory of Innate Immunity, Unit of Molecular Pathology, Biochemistry, and Immunology, Istituto di Ricovero e Cura a Carattere Scientifico (IRCCS) MultiMedica, 20138 Milan, Italy; matteo.gallazzi@multimedica.it (M.G.); barbara.bassani@multimedica.it (B.B.); 3 Department of Medicine and Innovative Technology, University of Insubria, 21100 Varese, Italy; mario.raspanti@uninsubria.it (M.R.); pieroantonio.zecca@uninsubria.it (P.A.Z.)

**Keywords:** dental pulp mesenchymal stem cells, nanostructured scaffold, angiogenesis, M2 polarization

## Abstract

Among all strategies directed at developing new tools to support re-vascularization of damaged tissues, the use of pro-angiogenic soluble factors, derived from mesenchymal stem cells (MSCs), appears a promising approach for regenerative medicine. Here, we compared the feasibility of two devices, generated by coupling soluble factors of human dental pulp mesenchymal stem cells (DPSCs), with a nanostructured scaffold, to support angiogenesis once transplanted in mice. DPSCs were obtained from impacted wisdom tooth removal, usually considered surgical waste material. After 28 days, we verified the presence of active blood vessels inside the scaffold through optical and scansion electron microscopy. The mRNA expression of surface antigens related to macrophage polarization (CD68, CD80, CD86, CD163, CD206), as well as pro-angiogenic markers (CD31, CD34, CD105, Angpt1, Angpt2, CDH5) was evaluated by real-time PCR. Our results demonstrate the capability of DPSC–scaffold and DPSC soluble factors–scaffold to support angiogenesis, similarly to adipose stem cells, whereas the absence of blood vessels was found in the scaffold grafted alone. Our results provide evidence that DPSC-conditioned medium can be proposed as a cell-free preparation able to support angiogenesis, thus, providing a relevant tool to overcome the issues and restrictions associated with the use of cells.

## 1. Introduction

Three-dimensional nanostructured scaffolds can play a crucial role in supporting new tissue formation by actively interacting with stem cells due to their capabilities to mimic extracellular matrix composition and its chemical/physical properties [1,2,3,4]. It was initially assumed that mesenchymal stem cells (MSCs) would engraft after being administered with biocompatible collagen-based scaffold, and differentiate into functional cells, resulting in the regeneration of tissues [5,6,7]. Instead, stem cells were demonstrated to inadequately engraft damaged tissues, in terms of number and/or time, to sustain efficient tissue repair and regeneration [8]. For this reason, in the last decades the research has been focused on formulating natural and synthetic 3D matrices that would allow the correct phenotypic switch, homing, and successful grafting through cell surface receptors and/or by paracrine route [9,10]. Following an injury, stromal cells were found remodeling the surrounding extracellular matrix, directly and/or via soluble factors, leading to a peculiar microenvironment that orchestrated cellular processes necessary to restore the physiological conditions [11].

In adults, angiogenesis represents a crucial step in several physiological conditions such as the female reproductive cycle, or the increase in blood influx during intense exercise and wound-healing processes [12,13,14]. It was established that the paracrine pathway serves as the primary mechanism to facilitate vessel sprouting, and the use of MSCs, as direct cell source, is not mandatory and may potentially introduce additional unpredictable occurrences. This scenario provides the rationale to employ cell-free derivatives, such as cell-conditioned media (here referred to also as secretome) composed of growth factors, soluble factors, and cytokines secreted by cells with pro-angiogenic features [15,16,17,18]. These factors play a pivotal role in controlling the proliferation, adhesion, and differentiation of stem and progenitor cells, as well as contributing to the establishment of fully functional vascular networks, essential during tissue regeneration [15,16,17,18].

It is well-known that angiogenesis can be sustained by both endothelial and non-endothelial cells, such as stromal cells (MSCs and fibroblasts) [18,19,20,21] and immune cells (innate and adaptive immunity). The capability of stromal and immune cells to undergo the angiogenic switch is finely tuned by the peculiar cytokine and chemokine milieu, characterizing the pathophysiological micro- (tissue) and macro- (peripheral blood) environment of the host organism and driving the so-called polarization state [22,23,24,25,26,27,28]. In this scenario, macrophages represent the most investigated immune cell type, found to be crucial in supporting angiogenesis during the repairing process. Nevertheless, the angiogenic switch has been documented for almost all cells from innate and adaptive immunity [29,30,31,32].

As mentioned, an efficient vascularization is necessary to support tissue regeneration of the injured area; for this reason, several strategies were developed, involving the use of MSC–secretome, extracellular matrix component, and biochemical cues [4,7,8,9,10,15,16,17,18,33,34]. MSCs represent the ideal source to obtain the cell–secretome, since they can be easily isolated and maintained in starvation up to 72 h [17,35]. Recently, human dental pulp mesenchymal stem cells (DPSCs) have emerged as a preferred source of MSCs; this is due to their higher proliferation rate and stemness features, compared to other types of MSCs, as well as the fact that they are obtained from the removal of impacted wisdom tooth, considered surgical waste material [36,37,38,39].

Here, we investigated in vivo the pro-angiogenic potential of a device, composed of DPSC–secretome and commercial Integra^®^ flowable wound matrix (FWM), in supporting angiogenesis, following 28 days engraftment in athymic BALB/c nude mice.

We found DPSC–secretome, here named conditioned medium (CM), was efficient in inducing the generation of mature blood vessels and in supporting the infiltration of pro-angiogenic M2 macrophages, as well as the DPSC cellular preparation, thus, demonstrating that the system supports angiogenesis similar to what is already observed with adipose stem cells (ASCs) [16,34].

Finally, the development of a cell-free device able to trigger the development of vascular networks, and their correct integration into tissues, is pivotal for regenerative medicine.

## 2. Materials and Methods

### 2.1. Scaffold

Integra^®^ flowable wound matrix (FWM) is a 3D porous biocompatible matrix composed of granulated cross-linked bovine tendon collagen and glycosaminoglycan, provided, and characterized by LifeSciences Corporation (Plainsboro, NJ, USA). Fibers have 2 μm thickness and 120 μm length, characterized by an average inter-fiber distance of 45 μm.

This device is commonly used for the treatment of tunneled and irregular wounds, which are often associated with excessive scar tissue formation. The dried-supplied product must be re-hydrated with culture media, where the scaffold acquires a jelly-like texture, ideal for subcutaneous injection. More information about the structure, porosity, and biocompatibility are available on the Integra website (www.integralife.com, accessed on 31 March 2021).

### 2.2. Cell Isolation and Maintenance

DPSCs were obtained from processed dental pulp of an impacted wisdom tooth of a young subject (15 years old), after surgical removal and the signature of informed consent. Cells were isolated according to the Gronthos protocol [40], modified in our laboratory. Briefly, the dental pulp was digested through collagenase type II (Sigma Aldrich, Milano, Italy) at 37 °C for 1 h, under agitation. The obtained fraction was filtered (70 μm cell strainers) and centrifuged at 960× *g* for 10 min (Neya 8—Remi Elektrotechnik Ltd., Mumbai, India); the resulting pellet was then cultured in T25 flasks at 37 °C, 5% CO_2_, in DMEM:DMEM F12 1:1 (Sigma Aldrich, Milano, Italy), supplemented with 2 mM L-Gln, 1% penicillin–streptomycin, 0.1% gentamicin, and 10% FBS. After 24 h, unattached cells were removed. All planned experiments required a huge number of DPSCs; thus, cells were subsequently cultured in T75 flasks until passage 5, which is considered as an early passage [41].

### 2.3. Conditioned Medium Preparation and Characterization

CM was obtained as described in Marcozzi et al. [42]. Briefly, once cells reached 70–80% of confluency, cell medium was removed and, following two washes in PBS 1X, cells were incubated for 48 h in FBS-free DMEM. The medium was then collected and centrifuged at 2000× *g* for 10 min to remove all cell fragments. To maximize protein content, the CM was concentrated using the Amicon Ultra 15 mL centrifugal filter device (Millipore, Darmstadt, Germany), with a 3 kDa cut-off, following manufacturer’s instructions. The concentrated media were collected and stored at −80 °C until use.

CM characterization was performed by enzyme-linked immunosorbent assay (ELISA), as reported in Barone et al. 2022, following the manufacturer’s instructions (FineTest^®^, Wuhan, China), to investigate the concentration of vascular endothelial growth factor A (VEGFA), hypoxia-inducible factor-1α (HIF-1α), and transforming growth factor-β (TGF-β).

The total amount was determined through the absorbance recording at 450 nm using the GloMax^®^ Discover Microplate Reader (Promega, Milano, Italy).

Each experiment was repeated 3 times and the obtained values were expressed as pg/mL.

### 2.4. In Vivo FWM Assay

The animal studies were approved by the University of Insubria Ethical Committee and by the Italian Ministry of Health, in accordance with the Italian D.Lgs 26/2014. Five seven-week-old male athymic BALB/c nude mice (Crl:CD1-Foxn1nu086) were purchased from Charles River (Calco, Lecco, Italy).

Mice were housed in the animal facility with 12 h light/dark cycles and fed ad libitum. Experiments were performed following the Italian and European Community guidelines (D.L. 2711/92 No. 116; 86/609/EEC Directive), the 3 Rs declaration, and within an approved protocol by the institutional ethics committee.

The grafting procedure was performed on mice under isoflurane anesthesia through a 0.5 cm incision on the dorsal side of the mice to introduce the scaffolds between the muscle and subcutaneous layer, using a syringe equipped with a Luer lock connector and a flexible injector.

The grafts consisted of the following formulations: (1) Integra^®^ FWM hydrated with fresh culture medium; (2) Integra^®^ FWM hydrated with fresh culture medium containing 3 × 10^6^ DPSCs; (3) Integra^®^ FWM hydrated with CM derived from 3 × 10^6^ DPSCs. Each syringe contained a total volume of 3 mL of each formulation and a volume of 200 μL, for each preparation, was injected in any single animal. Incisions were then stitched using surgical sterile strips.

### 2.5. Gross Examination of Scaffold

Following 28 days from the grafting, mice were sacrificed in a CO_2_ chamber. The scaffolds were harvested, observed by a circular lens (Canon EOS 550 D, Tokyo, Japan), and parameters such as dimension, color, consistency, and vascularization rate were recorded for each condition, and images captured.

### 2.6. Sample Collection

For microscopy observations, a small section of each sample was fixed in 4% PFA solution at RT for 2 h and preserved in 70% EtOH.

For molecular analysis, samples were stored at −80 °C until RNA extraction.

### 2.7. Microscopy Analysis and Blood Vessel Assessment

To evaluate the formation of new vessels in the inner portion of the scaffold, fixed samples were embedded in paraffin, following sequential dehydration with ethanol (70, 80, 90, 95, 100%), and cut using an RMC-RM3 rotary microtome (TiEsseLab, Milan, Italy). Five nonconsecutive sections (5 μm) per sample were placed on glass slides, then stained with hematoxylin and eosin (H&E) solution, following classical procedures, and finally analyzed. Vessels were counted considering the number of capillaries present in three-microscope fields of each sample, and then measured, recording the smaller diameter, using ISCapture software (version 3.6.9.4). According to their size the capillaries were classified as large (d > 100 µm), medium (100 < d > 20 µm), and small (d < 20 µm).

For SEM observation, specimens were dehydrated in graded ethanol and dried in hexamethyldisilazane (Sigma Aldrich, Milano, Italy), placed on aluminum stubs, then covered with 10 nm gold (Emitech K550) and observed with a Philips SEM-FEG XL-30 (Eindhoven, The Netherlands).

### 2.8. RNA Extraction, Reverse Transcription, and Real-Time PCR

Trizol extraction was performed to purify the total RNA, which was quantified by the QuantiFluor^®^ RNA System (Promega, Milano, Italy) and its integrity was assessed by 1% agarose gel electrophoresis. The iScript™ cDNA Synthesis Kit (BioRad, Milano, Italy) allowed the RNA transcription, and the obtained cDNA was stored at −20 °C until use. qPCR was performed using iTaq Universal SYBR^®^ Green Supermix (BioRad, Milano, Italy) and specific genes involved in angiogenesis, such as platelet endothelial cell adhesion molecule/cluster of Differentiation 31 (*Pecam1*/*CD31*), cluster of differentiation 34 (*CD34*), endoglin/cluster of differentiation 105 (*CD105*), angiopoietin 1 (*Angpt1*), angiopoietin 2 (*Angpt2*), and cadherin 5 (*CDH5*). Furthermore, the expression of macrophage polarization markers (*CD80*, *CD86*, *CD163*, *CD206*) was also evaluated. The Beacon Designer Program (BioRad, Milano, Italy) allowed the design of primers used for this experimental plan (sequences are shown in Table 1).Each sample was prepared as reported in Rossi et al. [43]. Briefly, 1 μL (5 ng) of cDNA, 1 μL of forward and reverse primer mix (6 μM), 7.5 μL of SYBR Green Supermix (2×), and water to a final volume of 15 μL were mixed and placed in the CFX 96 Thermocycler (BioRad, Milano, Italy). Values were normalized with two reference genes, glyceraldehyde phosphate dehydrogenase (*GAPDH*) and β-actin, according to the method of Palombella et al. [44] and quantified by using the ΔCt method. Each experiment was repeated three times.

### 2.9. Flow Cytometry

Following surgical excision from mice, engrafted Integra scaffolds were collected and placed in PBS for further processing. Scaffolds were subjected to enzymatic digestion using a type II collagenase solution (3 mg/mL), for one hour at 37 °C, under agitation every 10 min. Recovered material was then filtered, using a 100 μm pore cell strainer (BD Biosciences, Franklin Lakes, NJ, USA) prior to antibody staining and subsequent flow cytometry analysis. The single cell suspension was stained for 30 min, at 4 °C, in the dark, with the following monoclonal antibodies: FITC-CD31 (clone: MEC 13.3), BUV-395-CD45 (clone: 30-F11), PE-CF594-F4/80 (clone: T45-2342), BV-421-CD80 (clone: 16-10A1), Alexa Fluor-647-CD206 (clone: MDR5D3). Cells were washed and resuspended in PBS, then used for FACS analysis using a 5-lasers BD FACS Fortessa cell analyzer. Following single cell (based forward scatter area/FSC-A vs. forward scatter height/FSC-H, then side scatter area/SSC-A vs. side scatter height/SSC-H) and morphology gating (forward scatter area/FSC-A vs. side scatter area/SSC-A), the subset cell populations were identified as follows: CD45^−^ cells (stromal cells), CD45^−^CD31^+^ cells (endothelial cells), CD45^+^ cells (total leukocytes), CD45^+^F4/80^+^ cells (total macrophages), CD45^+^F4/80^+^CD80^+^ cells (M1-like macrophages), and CD45^+^F4/80^+^CD206^+^ (M2-like macrophages). FACS data were acquired with the FACS Diva software v8.0.1 (BD Biosciences) and analyzed using the FlowJo v10 software (BD Biosciences).

### 2.10. Statistical Analysis

Vessel count and classification are shown as mean of vessel/mm^2^ ± standard error. qPCR data statistical analysis was performed through Student’s *t*-test over the ΔCt values of scaffold supplied with the formulation (see Section 2.5 Xenogenic grafting) versus scaffold suspended in fresh culture medium, used as control. Data were expressed as mean values (±standard error); *p* values of *p* < 0.05 were considered statistically significant.

## 3. Results

### 3.1. Characterization of DPSCs Prior to In Vivo Engraftment

**Figure 1:**
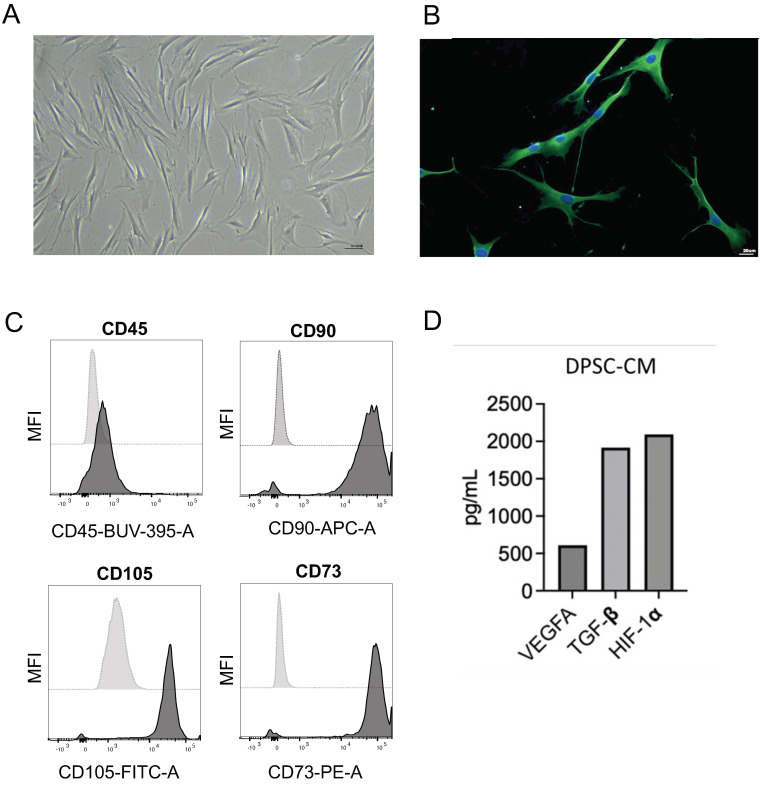
Characterization of DPSCs. DPSCs, isolated from an impacted wisdom tooth of a young subject (15 years old) were characterized by morphological inspection magnification 10×, scale bar 55.5 μm (**A**), immunofluorescence for CD44 signal, magnification 10×, scale bar 20 μm (**B**), flow cytometry for CD45 (pan-leukocyte marker), CD90, CD105, CD73 (as MSC surface antigen markers) (**C**), and ELISA (**D**).

We characterized the DPSCs, isolated from impacted wisdom tooth of a young subject (15 years old) by immunofluorescence (IF), flow cytometry, and ELISA. Isolated cells show the typical morphology of mesenchymal cells, as illustrated in Figure 1A.

The MSC identity was also confirmed by IF analysis for CD44 (Figure 1B). FACS analysis shows a total absence of CD45^+^ cells (as readout on potential leucocytes contamination) and bright signal intensity for CD90, CD105, and CD73, as standard MSC surface antigen markers (Figure 1C). Finally, we detected secretion of VEGFA, TGF-β, and HIF-1α, in DPSC–CM, as revealed by ELISA analysis (Figure 1D).

### 3.2. CM from DPSCs Efficiently Induces a Vascular Network In Vivo

**Figure 2:**
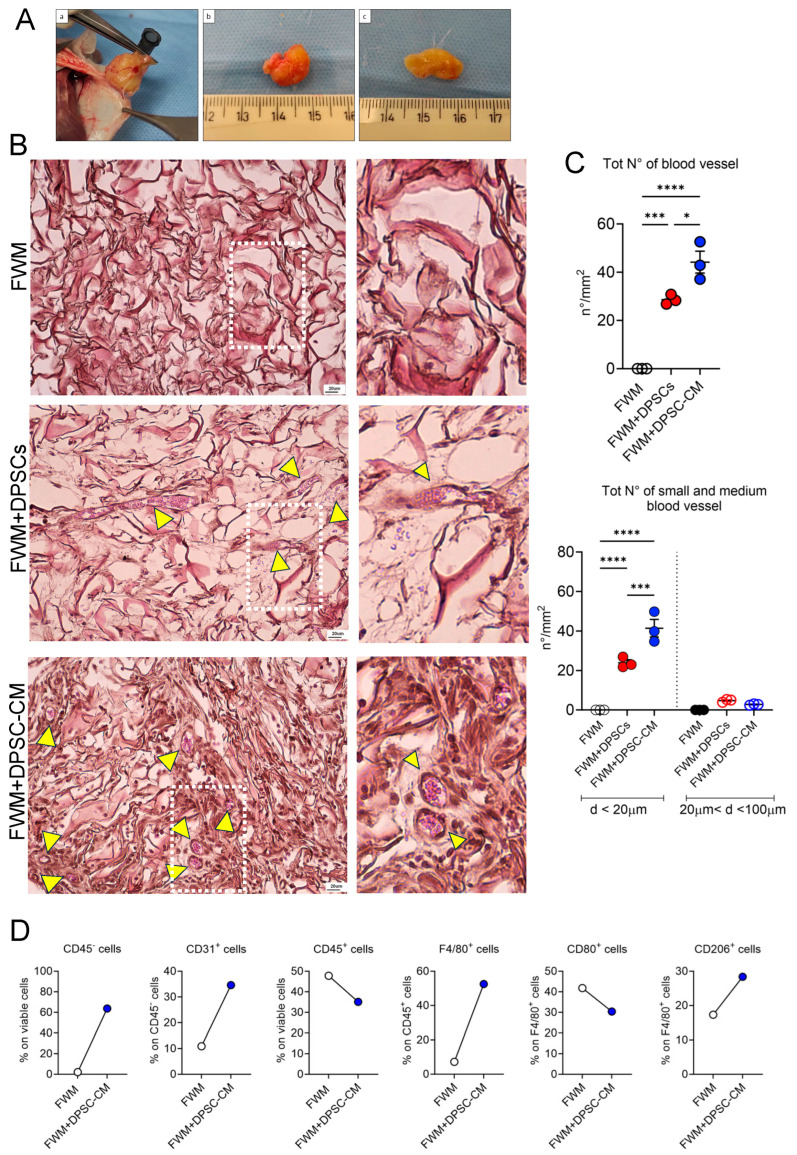
Pro-angiogenic effects of DPSCs and DPSC–CM in vivo. (**A**) Representative images of the whole observation of the removed scaffolds (**a**). The newly formed vascular network, spreading inside the scaffold, are visible in both preparations FWM and DPSCs (**b**), and FWM and DPSC–CM (**c**). (**B**) Representative microscopic images of scaffold specimens stained with H&E. FWM alone, FWM combined with DPSCs, and FWM combined with DPSC–CM. As indicated by the yellow arrows, numerous capillaries, full of erythrocytes, can be observed. Magnification 10×, scale bar 20 μm. (**C**) Total vessel count in the scaffolds, and subclassification as small and medium blood vessels are shown as number/mm^2^, (*n* = 3). (**D**) FACS analysis for cell infiltration in the scaffold alone (FWM) or supplemented with DPSC–CM for non-leukocytic cells (CD45^−^ cells), endothelial cells (CD31^+^ cells), total leukocytes (CD45^+^ cells), total macrophages (F4/80^+^ cells), M1 macrophages (CD80^+^ cells), M2 macrophages (CD206^+^ cells). Results are shown as mean ± SEM, one-way ANOVA, * *p* ≤ 0.05, *** *p* ≤ 0.001, **** *p* ≤ 0.0001.

To evaluate the vascularization rate of the implanted scaffolds, based on the presence of DPSCs or DPSC–CM, we performed integrated analysis by histology, flow cytometry, and ultrastructural analysis by SEM microscopy. Following 28 days from the grafting, the gross evaluation of the removed scaffolds did not show macroscopically differences in size, color, consistency, and vascularization, by comparing FWM and DPSCs vs. FWM and DPSC–CM samples (Figure 2A).

As shown by micrographs in Figure 2B, in FWM and DPSCs and FWM and DPSC–CM, in addition to collagen fibers and fibroblast nuclei, the presence of numerous capillaries full of erythrocytes can be clearly observed. Vessel count shows a statistically significant difference in the number of capillaries that is increased in FWM and DPSC–CM, compared to FWM and DPSCs (Figure 2C). Also, by checking for small and medium size vessel presence in our samples, we detected a statistically significant increased number of small size vessel in FWM and DPSC–CM, compared to FWM and DPSCs (Figure 2C). We did not find capillaries larger than 100 μm (Figure 2C). Apart from the presence of some fibroblasts, no blood vessels were found in the FWM alone (Figure 2B,C).

We also performed flow cytometry analysis, to further verify the presence of pro-angiogenic cells in the scaffold, associated with FWM and DPSC–CM, compared to the scaffold alone, following recovery from mice (Figure 2D). Scaffolds with FWM and DPSC–CM were highly infiltrated by different cell types, including stromal cells (CD45− cells) and leukocytes (CD45^+^ cells) (Figure 2D). By further characterizing the leukocytes present in the FWM and DPSC–CM, we found an enrichment of pf F4/80 macrophages that were mostly CD206^+^ M2 macrophages, rather than CD80^+^ M1 macrophages. Finally, we observed higher infiltration of CD31^+^ endothelial cells in FWM and DPSC–CM, which, together with CD206^+^ macrophages, represent cells with pro-angiogenic activities.

**Figure 3:**
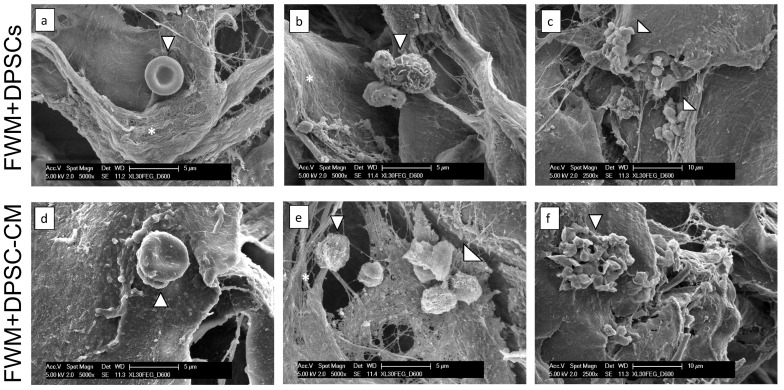
Ultrastructural analysis. Representative SEM images of FWM and DPSCs (**a**–**c**) and FWM and DPSC–CM (**d**–**f**) after 28 days from grafting. Newly formed collagen fibrils, indicated with *, are observed (**a**,**b**,**e**). Erythrocytes (**a**,**d**), leukocytes (**b**,**e**), and platelets (**c**,**f**), present both in FWM and DPSCs and FWM and DPSC–CM are indicated with arrowheads.

We then performed ultrastructural analysis of scaffolds containing DPSCs and DPSC–CM, by scanning electron microscopy (SEM) (Figure 3).

SEM images support the de novo collagen fibril deposition, generated by fibroblasts that colonize the scaffold. In both formulations, FWM and DPSCs and FWM and DPSC–CM, the presence of erythrocytes (Figure 3a,d), leucocytes (Figure 3b,e), and platelets (Figure 3c,f) corroborates our previous results regarding the vascularization occurring inside the scaffold by adipose-derived mesenchymal stem cells and their CM [5,8,21].

### 3.3. Scaffolds with DPSCs or DPSC–CM Show Similar Pro-Angiogenic Signatures at Gene Expression Level

To better map the presence of pro-angiogenic cell population/factors in our scaffolds, we performed a qPCR analysis, in the bulk material recovered from scaffolds.

**Figure 4:**
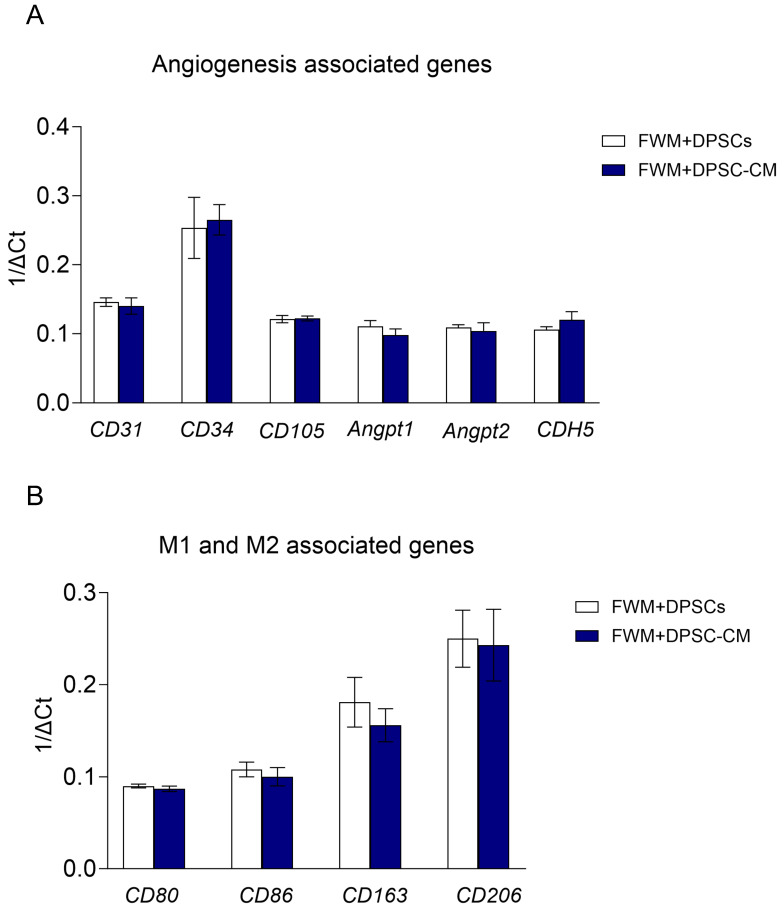
Real-time PCR analysis for pro-angiogenic cell populations (**A**) and for macrophage polarization state (**B**). No significant differences were observed *n* = 5; *p* < 0.05.

We observed a similar expression profile for the pro-angiogenic signature *CD31*, *CD34*, *CD105*, *Angpt1*, *Angpt2*, and *CDH5* (Figure 4A) in both formulations (FWM and DPSCs and FWM and DPSC–CM). Similarly, we also found an enrichment of those genes associated with M2 macrophage polarization, *CD163* and *CD206*, compared to those genes related to M1 macrophage polarization, *CD80* and *CD86* (Figure 4B).

## 4. Discussion

Tissue engineering, defined as the multidisciplinary field within regenerative medicine that combines biology, engineering, and materials science to design and develop functional biological substitutes, is now considered as the most promising approach to restore, repair, or enhance the function of damaged or diseased tissues and organs [31]. Major devices employed in tissue engineering include biocompatible nanostructured scaffolds that, mimicking extracellular matrix composition, can play a crucial role in supporting new tissue formation [1,2,3,4,5].

Since the generation of functional blood vessels is crucial to assure an efficient tissue regeneration or repair, even more increasing efforts have been addressed to promote the vascularization of damaged areas [45,46,47,48].

For all these reasons, researchers focused their attention on the development of devices consisting of bio-scaffolds, in combination cellular products, such as MSCs or, even better, their cellular derivatives such as the conditioned medium [1,2,3,4,5,8,10,15,16,17,34,35].

Here, we reported the pro-angiogenic potential, in vivo, of two devices, composed of the commercial Integra^®^FWM associated with DPSCs or with DPSC–CM, in supporting vascularization and infiltration of different cells with pro-angiogenic activities, following 28 days of engraftment in athymic BALB/c nude mice.

VEGFA, TGF-β, and HIF-1α, secreted by DPSCs, are biomolecules with a crucial role in the angiogenetic process. More precisely, VEGFA is implied in the early phase of angiogenesis, promoting the proliferation and migration of endothelial cells, whereas TGF-β is involved in vessel maturation [36,37]. These results clearly provide the rational to propose DPSCs as a source of pro-angiogenic factors (DPSC–CM).

To functionally demonstrate the pro-angiogenic activities of DPSCs, we compared the capabilities of the Integra^®^FWM, associated with DPSCs or DPSC–CM, to induce the generation of a vascular network in vivo.

Following 28 days from the graft, by microscopic inspection, we observed that blood vessels were absent in the Integra^®^FWM grafted alone; differently and as expected, the scaffolds loaded with DPSCs and DPSC–CM resulted in high vascularization, and the capillaries were full of erythrocytes. These results were also confirmed by FACS analysis that showed increased presence of CD31^+^ endothelial cells in FWM and DPSC–CM, compared to the scaffold alone. Long et al. claimed that the collagen presents in the scaffold, together with the endogenous VEGF, participates in organizing vessel sprouting [49]; the absence of vascularization found in our samples, suggests the inadequate amount of endogenous VEGF to promote vessel formation inside the scaffold. Ultrastructural SEM analysis confirmed the optical microscopy results and our hypothesis that newly deposited collagen fibrils occurred by fibroblasts that colonized the scaffold [5,21]; these results were also confirmed by the presence of CD45^−^, identifying stromal cell components, which were increased in the FWM and DPSC–CM, compared to the scaffold alone. Both histological and SEM analysis show that FWM and DPSCs and FWM and DPSC–CM are infiltrated by erythrocytes, platelets, and leukocytes. These results are similar to those from our previous results obtained on ASCs and their CM [4,16,34].

FACS analysis confirmed the increased presence of F4/80^+^ macrophages in the scaffold with DPSC–CM, compared to the scaffold alone. Of note, most of the F4/80^+^ macrophages in the DPSC–CM scaffold have a CD206^+^M2 polarization, characterizing pro-angiogenic macrophages.

We, therefore, performed an overall gene expression analysis, by quantitative PCR, on bulk cellular material recovered from FWM and DPSCs and FWM and DPSC–CM samples, and we found comparable expression of different pro-angiogenic markers, namely, *CD31*, *CD34*, *CD105*, *Angpt1*, *Angpt2*, and *CDH5* in both FWM and DPSCs and FWM and DPSC–CM.

Expression of CD31, also known as platelet endothelial cell adhesion molecule 1, is strongly expressed on the surface of mature endothelial cells and considered an appropriate marker to monitor vessel density in tissues. Taken together, our results support the hypothesis that observed capillaries were mature and functional in loaded scaffolds.

CDH5, also known as vascular endothelial cadherin, is involved in vascular development and survival, maintaining the endothelial cell contacts through its interaction with VEGF [50].

CD34, a marker for human hematopoietic stem cells, is also found in tip cells and peculiar cell population protruding out from existing vascular structures that are the leading cells contributing to the generation of new vessels, by sprouting angiogenesis [51].

CD105, a cell membrane glycoprotein, is the most important marker for MSCs [52]; however, CD105 acts as an accessory receptor for TGF-β [53,54,55], found overexpressed in actively proliferating endothelial cells, and it is considered as a powerful marker of new vessel formation. Together with CD34, CD105 can also be considered a marker for early stages of vascularization.

Angiopoietins constitute an important family of growth factors, whose actions are mediated by Tie1 and Tie2 phosphorylation. The best characterized are angiopoietin-1 (Angpt1) and angiopoietin-2 (Angpt2). Similarly, to CDH5, Angpt1 is involved in vascular remodeling and protection through tightening of endothelial cell junctions [56]. Conversely, Angpt2 was initially identified as a vascular disruptive agent with antagonistic activity; however, recent data demonstrate that Angpt2 may have context-dependent agonist activities, as demonstrated in several conditions, such as the absence of Tie2 [57] or Angpt1 [58]. Furthermore, Xie et al. demonstrated the presence of Angpt2 on human hepatocellular carcinoma-derived exosomes and it was delivered into human umbilical vein endothelial cells via exosome endocytosis to stimulate angiogenesis by a Tie2-independent pathway [59]. This being the case, the mRNA expression of *Angpt2*, found in both formulations, supports the idea that these preparations could be favorable in promoting angiogenesis. qPCR also demonstrated that FWM and DPSCs and FWM and DPSC–CM samples are characterized by a similar enrichment in genes encoding for a M2/pro-angiogenic macrophage signature, rather than M1/pro-inflammatory angiogenic macrophage signatures.

A limitation of our study deals with a more exhaustive characterization of the soluble factors presents in the CM. We previously reported that CM from another type of MSCs, namely adipose MSCs (ASCs), were able to support angiogenesis in vitro and in vivo, similarly to the effects exerted by MSCs, as whole cells [16,34]. We recently characterized the CM of ASCs and we found a tremendous enrichment of pro-angiogenic soluble factors, strongly increased by hypoxic conditions [16]. Here, we already found, by ELISA assay, that VEGF, TGF-β, and HIF-1α, as major soluble pro-angiogenic factors, are produced by DPSC–CMs. These results were corroborated by the pro-angiogenic activities of DPSC–CM, associated with FWM in vivo. Another intriguing point is whether the soluble factors present in the DPSC–CM act alone or together with microvesicle-encapsulated factors. This point, together with a better characterization of CMs, will be part of our oncoming work. Also considering these limitations, our results are very encouraging, since they support the cell-free approach for regenerative medicine, and give an immediate positive functional readout, namely, pro-angiogenic activity.

Finally, as future perspectives, our results provide the rationale to employ DPSC–CM as a source of soluble factors to be potentially translated to clinical approaches in regenerative medicine aimed at restoring the damaged/compromised vasculature.

## 5. Conclusions

The development of a cell-free device able to guide the development of a new vascular network still represents a challenging urgency for regenerative medicine. The combination of biocompatible scaffolds with DPSC-derived soluble factors present in conditioned media (DPSC–CMs) has substantial advantages, compared to the direct employments of stem cells. This allows for the overcoming of different critical issues that range from cell handling and maintenance, storage, and standardization, thus, making DPSC–CM formulation as a promising biopharmaceutical product. The use of a combination of growth factors, such as those present in DPSC–CM, could be more advantageous and cost-effective compared to the addition of single growth factors.

In line with these critical issues, we demonstrated that our DPSC–CM formulation has the same pro-angiogenic features, in term of induction of a vascular network and infiltration of endothelial and non-endothelial pro-angiogenic cells, thus, outlining the possibility to employ DPSC–CM as a soluble factor formulation for regenerative medicine application with a cell-free approach.

## Figures and Tables

**Table 1:** List of primer sequences.

Gene Name	Sequence 5′-3′	T_m_ (°C)	Accession Number
***Mm_β-Actin***	*Fw* GCCCAGAGCAAGAGAGGTA *Rv* TAGAAGGTGTGGTGCCAGAT	65.0	NM_007393.5
		64.9	
***Mm_GAPDH***	*Fw* ACCTGCCAAGTATGATGAC *Rv* GGAGTTGCTGTTGAAGTC	64.0	NM_008084.3
		59.7	
***Mm_CD80***	*Fw* TTATCATCCTGGGCCTGGTC *Rv* GTGTCTGCAGATGGGTTTCC	65.4	NM_001359898.1
		65.2	
***Mm_CD86***	*Fw* TGCTGCTCATCATTGTATGT *Rv* GGTTCAAGTTCCTTCAGGTT	61.5	NM_019388.3
		61.9	
***Mm_CD163***	*Fw* GGTGCTGGATCTCCTGGTTG *Rv* CAGGAGCGTTAGTGACAGCA	66.8	NM_001170395.1
		66.3	
***Mm_CD206***	*Fw* GGCTGATTACGAGCAGTGGA *Rv* CATCACTCCAGGTGAACCCC	66.2	NM_008625.2
		66.5	
***Mm_CD31***	*Fw* AACAGAGCCAGCAGTATGA *Rv* ATGACAACCACCGCAATG	62.6	NM_008816.3
		62.5	
***Mm_CD34***	*Fw* CTGCTCCGAGTGCCATTA *Rv* CTCCTCACAACTAGATGCTTCA	63.3	NM_133654.3
		63.7	
***Mm_CD105***	*Fw* CGATAGCAGCACTGGATGAC *Rv* TGGCAAGCACAAGAATGGT	64.7	NM_007932.2
		64.5	
***Mm_Angpt1***	*Fw* GGAAGATGGAAGCCTGGATT *Rv* ACTGCCTCTGACTGGTTATTG	65.1	NM_009640.4
		65.2	
***Mm_Angpt2***	*Fw* CGACTACGACGACTCAGT *Rv* TCTCCACCATCTCCTTCTTC	63.7	NM_007426.4
		63.8	
***Mm_CDH5***	*Fw* CAGAGTCCATCGCAGAGT *Rv* AGCCAGCATCTTGAACCT	64.1	NM_009868.4
		64.4	

*Fw*: forward primer; *Rv*: reverse primer.

## Data Availability

Raw data for the article are available, pending request to the corresponding authors.

## References

[1] Zhang Y., Wu D., Zhao X., Pakvasa M., Tucker A.B., Luo H., Qin K.H., Hu D.A., Wang E.J., Li A.J. (2020). Stem Cell-Friendly Scaffold Biomaterials: Applications for Bone Tissue Engineering and Regenerative Medicine. Front. Bioeng. Biotechnol..

[2] Krishna L., Dhamodaran K., Jayadev C., Chatterjee K., Shetty R., Khora S.S., Das D. (2016). Nanostructured scaffold as a determinant of stem cell fate. Stem Cell Res. Ther..

[3] Golchin A., Farzaneh S., Porjabbar B., Sadegian F., Estaji M., Ranjbarvan P., Kanafimahbob M., Ranjbari J., Salehi-Nik N., Hosseinzadeh S. (2021). Regenerative Medicine Under the Control of 3D Scaffolds: Current State and Progress of Tissue Scaffolds. Curr. Stem Cell Res. Ther..

[4] Borgese M., Barone L., Rossi F., Raspanti M., Papait R., Valdatta L., Bernardini G., Gornati R. (2020). Effect of Nanostructured Scaffold on Human Adipose-Derived Stem Cells: Outcome of In Vitro Experiments. Nanomaterials.

[5] Khan F., Tanaka M. (2017). Designing Smart Biomaterials for Tissue Engineering. Int. J. Mol. Sci..

[6] Huynh N.P.T., Brunger J.M., Gloss C.C., Moutos F.T., Gersbach C.A., Guilak F. (2018). Genetic Engineering of Mesenchymal Stem Cells for Differential Matrix Deposition on 3D Woven Scaffolds. Tissue Eng. Part A.

[7] Liu H., MacQueen L.A., Usprech J.F., Maleki H., Sider K.L., Doyle M.G., Sun Y., Simmons C.A. (2018). Microdevice arrays with strain sensors for 3D mechanical stimulation and monitoring of engineered tissues. Biomaterials.

[8] Cherubino M., Valdatta L., Balzaretti R., Pellegatta I., Rossi F., Protasoni M., Tedeschi A., Accolla R.S., Bernardini G., Gornati R. (2016). Human adipose-derived stem cells promote vascularization of collagen-based scaffolds transplanted into nude mice. Regen. Med..

[9] Yang B., Wei K., Loebel C., Zhang K., Feng Q., Li R., Wong S.H.D., Xu X., Lau C., Chen X. (2021). Enhanced mechanosensing of cells in synthetic 3D matrix with controlled biophysical dynamics. Nat. Commun..

[10] Su N., Gao P.L., Wang K., Wang J.Y., Zhong Y., Luo Y. (2017). Fibrous scaffolds potentiate the paracrine function of mesenchymal stem cells: A new dimension in cell-material interaction. Biomaterials.

[11] Rabelink T.J., Little M.H. (2013). Stromal cells in tissue homeostasis: Balancing regeneration and fibrosis. Nat. Rev. Nephrol..

[12] Carmeliet P. (2003). Angiogenesis in health and disease. Nat. Med..

[13] Carmeliet P., Jain R.K. (2000). Angiogenesis in cancer and other diseases. Nature.

[14] Carmeliet P. (2005). Angiogenesis in life, disease and medicine. Nature.

[15] Kumar P., Kandoi S., Misra R., Vijayalakshmi S., Rajagopal K., Verma R.S. (2019). The mesenchymal stem cell secretome: A new paradigm towards cell-free therapeutic mode in regenerative medicine. Cytokine Growth Factor Rev..

[16] Barone L., Palano M.T., Gallazzi M., Cucchiara M., Rossi F., Borgese M., Raspanti M., Zecca P.A., Mortara L., Papait R. (2023). Adipose mesenchymal stem cell-derived soluble factors, produced under hypoxic condition, efficiently support in vivo angiogenesis. Cell Death Discov..

[17] Han Y., Yang J., Fang J., Zhou Y., Candi E., Wang J., Hua D., Shao C., Shi Y. (2022). The secretion profile of mesenchymal stem cells and potential applications in treating human diseases. Signal Transduct. Target. Ther..

[18] Merino-Gonzalez C., Zuniga F.A., Escudero C., Ormazabal V., Reyes C., Nova-Lamperti E., Salomon C., Aguayo C. (2016). Mesenchymal Stem Cell-Derived Extracellular Vesicles Promote Angiogenesis: Potencial Clinical Application. Front. Physiol..

[19] Newman A.C., Nakatsu M.N., Chou W., Gershon P.D., Hughes C.C. (2011). The requirement for fibroblasts in angiogenesis: Fibroblast-derived matrix proteins are essential for endothelial cell lumen formation. Mol. Biol. Cell.

[20] Saraswati S., Marrow S.M.W., Watch L.A., Young P.P. (2019). Identification of a pro-angiogenic functional role for FSP1-positive fibroblast subtype in wound healing. Nat. Commun..

[21] Kalluri R., Zeisberg M. (2006). Fibroblasts in cancer. Nat. Rev. Cancer.

[22] Gieseck R.L., Wilson M.S., Wynn T.A. (2018). Type 2 immunity in tissue repair and fibrosis. Nat. Rev. Immunol..

[23] Minton K. (2019). Connecting angiogenesis and autoimmunity. Nat. Rev. Immunol..

[24] Bruno A., Pagani A., Pulze L., Albini A., Dallaglio K., Noonan D.M., Mortara L. (2014). Orchestration of angiogenesis by immune cells. Front. Oncol..

[25] Frantz S., Vincent K.A., Feron O., Kelly R.A. (2005). Innate immunity and angiogenesis. Circ. Res..

[26] Bhagwani A., Thompson A.A.R., Farkas L. (2020). When Innate Immunity Meets Angiogenesis-The Role of Toll-Like Receptors in Endothelial Cells and Pulmonary Hypertension. Front. Med..

[27] Varricchi G., Loffredo S., Galdiero M.R., Marone G., Cristinziano L., Granata F., Marone G. (2018). Innate effector cells in angiogenesis and lymphangiogenesis. Curr. Opin. Immunol..

[28] Ribatti D., Crivellato E. (2009). Immune cells and angiogenesis. J. Cell. Mol. Med..

[29] Parisi L., Gini E., Baci D., Tremolati M., Fanuli M., Bassani B., Farronato G., Bruno A., Mortara L. (2018). Macrophage Polarization in Chronic Inflammatory Diseases: Killers or Builders?. J. Immunol. Res..

[30] Cassetta L., Cassol E., Poli G. (2011). Macrophage polarization in health and disease. Sci. World J..

[31] Biswas S.K., Chittezhath M., Shalova I.N., Lim J.Y. (2012). Macrophage polarization and plasticity in health and disease. Immunol. Res..

[32] Sica A., Mantovani A. (2012). Macrophage plasticity and polarization: In vivo veritas. J. Clin. Investig..

[33] Yamada K.M., Doyle A.D., Lu J. (2022). Cell-3D matrix interactions: Recent advances and opportunities. Trends Cell Biol..

[34] Barone L., Rossi F., Valdatta L., Cherubino M., Papait R., Binelli G., Romano N., Bernardini G., Gornati R. (2022). Human Adipose-Derived Stem Cell-Conditioned Medium Promotes Vascularization of Nanostructured Scaffold Transplanted into Nude Mice. Nanomaterials.

[35] Gonzalez-Gonzalez A., Garcia-Sanchez D., Dotta M., Rodriguez-Rey J.C., Perez-Campo F.M. (2020). Mesenchymal stem cells secretome: The cornerstone of cell-free regenerative medicine. World J. Stem Cells.

[36] Costela-Ruiz V.J., Melguizo-Rodriguez L., Bellotti C., Illescas-Montes R., Stanco D., Arciola C.R., Lucarelli E. (2022). Different Sources of Mesenchymal Stem Cells for Tissue Regeneration: A Guide to Identifying the Most Favorable One in Orthopedics and Dentistry Applications. Int. J. Mol. Sci..

[37] Krivanek J., Soldatov R.A., Kastriti M.E., Chontorotzea T., Herdina A.N., Petersen J., Szarowska B., Landova M., Matejova V.K., Holla L.I. (2020). Dental cell type atlas reveals stem and differentiated cell types in mouse and human teeth. Nat. Commun..

[38] Li B., Ouchi T., Cao Y., Zhao Z., Men Y. (2021). Dental-Derived Mesenchymal Stem Cells: State of the Art. Front. Cell Dev. Biol..

[39] Ledesma-Martinez E., Mendoza-Nunez V.M., Santiago-Osorio E. (2016). Mesenchymal Stem Cells Derived from Dental Pulp: A Review. Stem Cells Int..

[40] Gronthos S., Mankani M., Brahim J., Robey P.G., Shi S. (2000). Postnatal human dental pulp stem cells (DPSCs) in vitro and in vivo. Proc. Natl. Acad. Sci. USA.

[41] Ratushnyy A., Ezdakova M., Buravkova L. (2020). Secretome of Senescent Adipose-Derived Mesenchymal Stem Cells Negatively Regulates Angiogenesis. Int. J. Mol. Sci..

[42] Marcozzi C., Frattini A., Borgese M., Rossi F., Barone L., Solari E., Valli R., Gornati R. (2020). Paracrine effect of human adipose-derived stem cells on lymphatic endothelial cells. Regen. Med..

[43] Rossi F., Bernardini G., Bonfanti P., Colombo A., Prati M., Gornati R. (2009). Effects of TCDD on spermatogenesis related factor-2 (SRF-2): Gene expression in Xenopus. Toxicol. Lett..

[44] Palombella S., Pirrone C., Cherubino M., Valdatta L., Bernardini G., Gornati R. (2017). Identification of reference genes for qPCR analysis during hASC long culture maintenance. PLoS ONE.

[45] Phelps E.A., Garcia A.J. (2010). Engineering more than a cell: Vascularization strategies in tissue engineering. Curr. Opin. Biotechnol..

[46] Masson-Meyers D.S., Tayebi L. (2021). Vascularization strategies in tissue engineering approaches for soft tissue repair. J. Tissue Eng. Regen. Med..

[47] Lovett M., Lee K., Edwards A., Kaplan D.L. (2009). Vascularization strategies for tissue engineering. Tissue Eng. Part B Rev..

[48] Dellaquila A., Le Bao C., Letourneur D., Simon-Yarza T. (2021). In Vitro Strategies to Vascularize 3D Physiologically Relevant Models. Adv. Sci..

[49] Fonsatti E., Sigalotti L., Arslan P., Altomonte M., Maio M. (2003). Emerging role of endoglin (CD105) as a marker of angiogenesis with clinical potential in human malignancies. Curr. Cancer Drug Targets.

[50] Sauteur L., Krudewig A., Herwig L., Ehrenfeuchter N., Lenard A., Affolter M., Belting H.G. (2014). Cdh5/VE-cadherin promotes endothelial cell interface elongation via cortical actin polymerization during angiogenic sprouting. Cell Rep..

[51] Siemerink M.J., Klaassen I., Vogels I.M., Griffioen A.W., Van Noorden C.J., Schlingemann R.O. (2012). CD34 marks angiogenic tip cells in human vascular endothelial cell cultures. Angiogenesis.

[52] Cho S.K., Bourdeau A., Letarte M., Zuniga-Pflucker J.C. (2001). Expression and function of CD105 during the onset of hematopoiesis from Flk1^+^ precursors. Blood.

[53] Jadhao M., Chen C.L., Liu W., Deshmukh D., Liao W.T., Chen J.Y., Urade R., Tsai E.M., Hsu S.K., Wang L.F. (2022). Endoglin Modulates TGFbetaR2 Induced VEGF and Proinflammatory Cytokine Axis Mediated Angiogenesis in Prolonged DEHP-Exposed Breast Cancer Cells. Biomedicines.

[54] ten Dijke P., Goumans M.J., Pardali E. (2008). Endoglin in angiogenesis and vascular diseases. Angiogenesis.

[55] Alt A., Miguel-Romero L., Donderis J., Aristorena M., Blanco F.J., Round A., Rubio V., Bernabeu C., Marina A. (2012). Structural and functional insights into endoglin ligand recognition and binding. PLoS ONE.

[56] Akwii R.G., Sajib M.S., Zahra F.T., Mikelis C.M. (2019). Role of Angiopoietin-2 in Vascular Physiology and Pathophysiology. Cells.

[57] Felcht M., Luck R., Schering A., Seidel P., Srivastava K., Hu J., Bartol A., Kienast Y., Vettel C., Loos E.K. (2012). Angiopoietin-2 differentially regulates angiogenesis through TIE2 and integrin signaling. J. Clin. Investig..

[58] Yuan H.T., Khankin E.V., Karumanchi S.A., Parikh S.M. (2009). Angiopoietin 2 is a partial agonist/antagonist of Tie2 signaling in the endothelium. Mol. Cell. Biol..

[59] Xie J.Y., Wei J.X., Lv L.H., Han Q.F., Yang W.B., Li G.L., Wang P.X., Wu S.B., Duan J.X., Zhuo W.F. (2020). Angiopoietin-2 induces angiogenesis via exosomes in human hepatocellular carcinoma. Cell Commun. Signal..

